# Reliability and validity testing of team emergency assessment measure in a distributed team context

**DOI:** 10.3389/fpsyg.2023.1110306

**Published:** 2023-04-20

**Authors:** Hanna Morian, Maria Härgestam, Magnus Hultin, Håkan Jonsson, Karin Jonsson, Torben Nordahl Amorøe, Johan Creutzfeldt

**Affiliations:** ^1^Department of Nursing, Umeå University, Umeå, Sweden; ^2^Department of Surgical and Perioperative Sciences, Anesthesia and Critical Care Medicine, Umeå University, Umeå, Sweden; ^3^Department of Epidemiology and Global Health, Umeå University, Umeå, Sweden; ^4^Department of Molecular and Clinical Medicine, Institute of Medicine, Sahlgrenska Academy, University of Gothenburg, Gothenburg, Sweden; ^5^Simulation Center West, Department of Research, Education, and Development, Region Västra Götaland, Sahlgrenska University Hospital, Gothenburg, Sweden; ^6^Department of Clinical Science, Intervention, and Technology, Karolinska Institute, Stockholm, Sweden; ^7^Center for Advanced Medical Simulation and Training, Karolinska University Hospital, Stockholm, Sweden

**Keywords:** interprofessional teams, team performance, teamwork, distributed team, telemedicine, instrument, validation, assessments

## Abstract

Medical multi-professional teams are increasingly collaborating *via* telemedicine. In distributed team settings, members are geographically separated and collaborate through technology. Developing improved training strategies for distributed teams and finding appropriate instruments to assess team performance is necessary. The Team Emergency Assessment Measure (TEAM), an instrument validated in traditional collocated acute-care settings, was tested for validity and reliability in this study when used for distributed teams. Three raters assessed video recordings of simulated team training scenarios (*n* = 18) among teams with varying levels of proficiency working with a remotely located physician *via* telemedicine. Inter-rater reliability, determined by intraclass correlation, was 0.74–0.92 on the TEAM instrument’s three domains of leadership, teamwork, and task management. Internal consistency (Cronbach’s alpha) ranged between 0.89–0.97 for the various domains. Predictive validity was established by comparing scores with proficiency levels. Finally, concurrent validity was established by high correlations, >0.92, between scores in the three TEAM domains and the teams’ overall performance. Our results indicate that TEAM can be used in distributed acute-care team settings and consequently applied in future-directed learning and research on distributed healthcare teams.

## 1. Introduction

With the increasing use of telemedicine, alternative team structures have emerged in healthcare (Butler et al., 2019). Telemedicine uses electronic information and communications technologies to provide and support healthcare when distance separates the participants (Field, 1996). The COVID-19 pandemic has brought telemedicine to the forefront of healthcare systems; today, telemedicine is widely used and highly relevant (Bains et al., 2020; Vilendrer et al., 2020; Garattini et al., 2021). By enabling local medical staff to be connected to specialists *via* a video link, telemedicine can assist in bringing first-rate healthcare to remote areas (Craig and Patterson, 2005).

A considerable amount of research has focused on understanding how teams work effectively (Salas et al., 2016). Teamwork is generally seen as more challenging in distributed team settings (Bolle et al., 2009); however, the added complexity needs to be defined. Distributed teams can be distinguished from traditionally collocated teams in terms of collaboration through communication technologies and their geographical dispersion (Bell and Kozlowski, 2002). Typically, they include knowledge workers with unique skills (Bell and Kozlowski, 2002). In addition, they can vary in structure, from entirely distributed teams when all team members are distributed to different locations to partially distributed teams, where the number and size of isolated and collocated subgroups differ (O'Leary and Cummings, 2007). Today, it is still being determined how technology and the lack of the physical presence of team members impact teamwork in healthcare teams.

Across healthcare, high-performing team functions are critical in providing safe patient care (Shapiro et al., 2008; Valentine et al., 2015). Analyses of human errors in medicine have revealed that poor teamwork skills are often at the heart of mistakes and failures (Kohn et al., 2000). Insufficient leadership, communication, decision-making, and collaboration (i.e., teamwork skills or non-technical skills) are associated with many adverse events, leading to patient injury, permanent disability, and even death (Boet et al., 2019). Team research has proven that education and training can improve team processes and patient safety outcomes (Weaver et al., 2014). In particular, increased training in non-technical skills enhances team performance (American College of Surgeons Committee on Trauma, 2013) and patient safety (Manser, 2009).

Instruments to evaluate team performance are essential to determine the effectiveness of team training, and instruments addressing crucial teamwork skills help foster clinicians’ understanding and guide training (Boet et al., 2019). Furthermore, a vital aspect of training effective teamwork is to include the team’s specific challenges and environment (Manser, 2009) when planning the training program since clinical context greatly affects how team members work together (Schmutz et al., 2019). Several instruments for performance evaluation have been developed and validated for teams working in traditional co-located settings, either in clinical or simulation-based environments (Valentine et al., 2015; Boet et al., 2019; Bhangu et al., 2022). Available instruments range in focus from assessing general teamwork skills (e.g., Healthy Teams Model; Mickan and Rodger, 2005) to more context-specific skills such as Non-Technical Skills for Surgeons (NOTSS; Yule et al., 2008). The increased use of telemedicine makes it necessary to validate instruments for assessing team performance in distributed teams, considering their profoundly different working conditions.

The Team Emergency Assessment Measure (TEAM) was developed by Cooper and colleagues as an instrument focusing on team performance specific to the cardiac resuscitation context (Cooper et al., 2010). The instrument was subsequently recognized as valid and reliable in several studies for emergency teams (Cant et al., 2016) in simulated and clinical settings with students (Hultin et al., 2019) and medical staff (Cooper et al., 2016). TEAM has also been translated into and validated for languages other than English (Maignan et al., 2016; Karlgren et al., 2021).

Even though TEAM is an established measurement of teamwork with good psychometric properties for emergencies (Valentine et al., 2015; Boet et al., 2019; Bhangu et al., 2022), to the best of our knowledge, its validity and reliability have not been established for the distributed team context. To address this gap, we report on the reliability and validity of TEAM for distributed teams managing acute medical conditions when the physician participates from a remote location *via* telemedicine.

## 2. Methods

### 2.1. Ethics

The Swedish Ethical Review Authority reviewed our application (registration number 2021-01027, date of decision: 2021-03-22). Since no intervention in a manner specified in Swedish legislation on ethics was planned, they concluded that this study was exempt from formal ethics approval. Nevertheless, the review authority presented no ethical objections to the study during the vetting process. Written informed consent was obtained, and the participants were informed that they were free to withdraw their consent without further explanation.

### 2.2. Data collection

Data collection consists of two stages, presented in chronological order: stage 1: simulation-based team training, and Stage 2: rating procedure.

#### 2.2.1. Stage 1: simulation-based team training

Data were collected in the autumn of 2021 at the Clinical Training Center at Umeå University in Northern Sweden during video-recorded simulation-based team training in which the physician participated remotely.

##### 2.2.1.1. Participants

A total of 27 participants were recruited: nine students (nursing and medical) at Umeå University and 18 medical staff (assistant nurses, registered nurses (RNs), and physicians) from the emergency department (ED) at Umeå University Hospital. Students referred to as *beginners* were invited during their final year of education through e-mail and classroom announcements and during digital seminars. ED managers, who were blinded to the study aim, invited medical staff with limited work experience in their field and/or ongoing specialist training, referred to as *intermediates*, and medical staff with extensive work experience in their field and/or specialists, referred to as *experts*. The participants were organized into nine three-person teams based on their proficiency level (Table 1): beginners (Teams 1–3), intermediates (Teams 4–6), and experts (Teams 7–9). At the beginner level, each team consisted of two student nurses (in the 5th or 6th semester) and one medical student (in the 10th or 11th semester). At the intermediate and expert levels, each team consisted of one assistant nurse, one RN, and one physician, according to standard practice in small emergency teams. In contrast to the other participants, the nursing and medical students had never worked together. In all teams, except one, both genders were represented. The characteristics of the study population are presented in Table 1. All participants completed the study.

**Table 1:** Study population characteristics.

	Participants	Teams 1–3	Teams 4–6	Teams 7–9
	All	Beginner	Intermediate	Expert
	*N* = 27	*N* = 9	*N* = 9	*N* = 9
Age median (Q1–Q3)	30 (25–43)	25 (24–27.5)	38 (29–46)	42 (28.5–52)
Female *n* (%)	17 (63)	6 (67)	5 (56)	6 (67)
Male *n* (%)	10 (37)	3 (33)	4 (44)	3 (33)
Nursing student *n* (%)	6 (22)	6 (67)	0 (0)	0 (0)
Medical student *n* (%)	3 (11)	3 (33)	0 (0)	0 (0)
Assistant nurse *n* (%)	6 (22)	0 (0)	3 (33)	3 (33)
Registered nurse *n* (%)	6 (22)	0 (0)	3 (33)	3 (33)
Physician *n* (%)	6 (22)	0 (0)	3 (33)	3 (33)
Work experience: year median (Q1–Q3)	4 (1.8–10.5)	1 (1–3)	5 (4–11)	11 (4.3–19.5)
No previous experience in team training, *n* (%)	1(4)^*^	0 (0)	1 (11)	0 (0)^*^
Previous experience in team training < 5 events, *n* (%)	11 (42)^*^	7 (78)	2 (22)	2 (25)^*^
Previous experience in team training ≥ 5 events, *n* (%)	14 (54)^*^	2 (22)	6 (67)	6 (75)^*^
Previous experience working in a distributed team, *n* (%)	4 (15)^*^	4 (44)	0 (0)	0 (0)^*^

^*^One missing value.

##### 2.2.1.2. Scenarios for team training

Each team participated in two scripted scenarios in which they were instructed to assess and treat a patient with deteriorating vital signs in the emergency room. For all teams, the patient suffered a urosepsis in the first scenario, whereas in the second scenario; the patient experienced a myocardial infarction. A standardized patient setup was used to support standardization and encourage interaction—more specifically, an individual was trained to follow a script to portray the patient (Felix and Simon, 2020). Each scenario was designed to last for about 20 min and displayed a medical emergency requiring immediate action. Furthermore, the scenarios were designed so that the patient’s condition would deteriorate at given times. The complexity and difficulty of the two scenarios were established beforehand by an expert group of experienced physicians and nurses in the area to make them comparable.

##### 2.2.1.3. Setting

**Figure 1:**
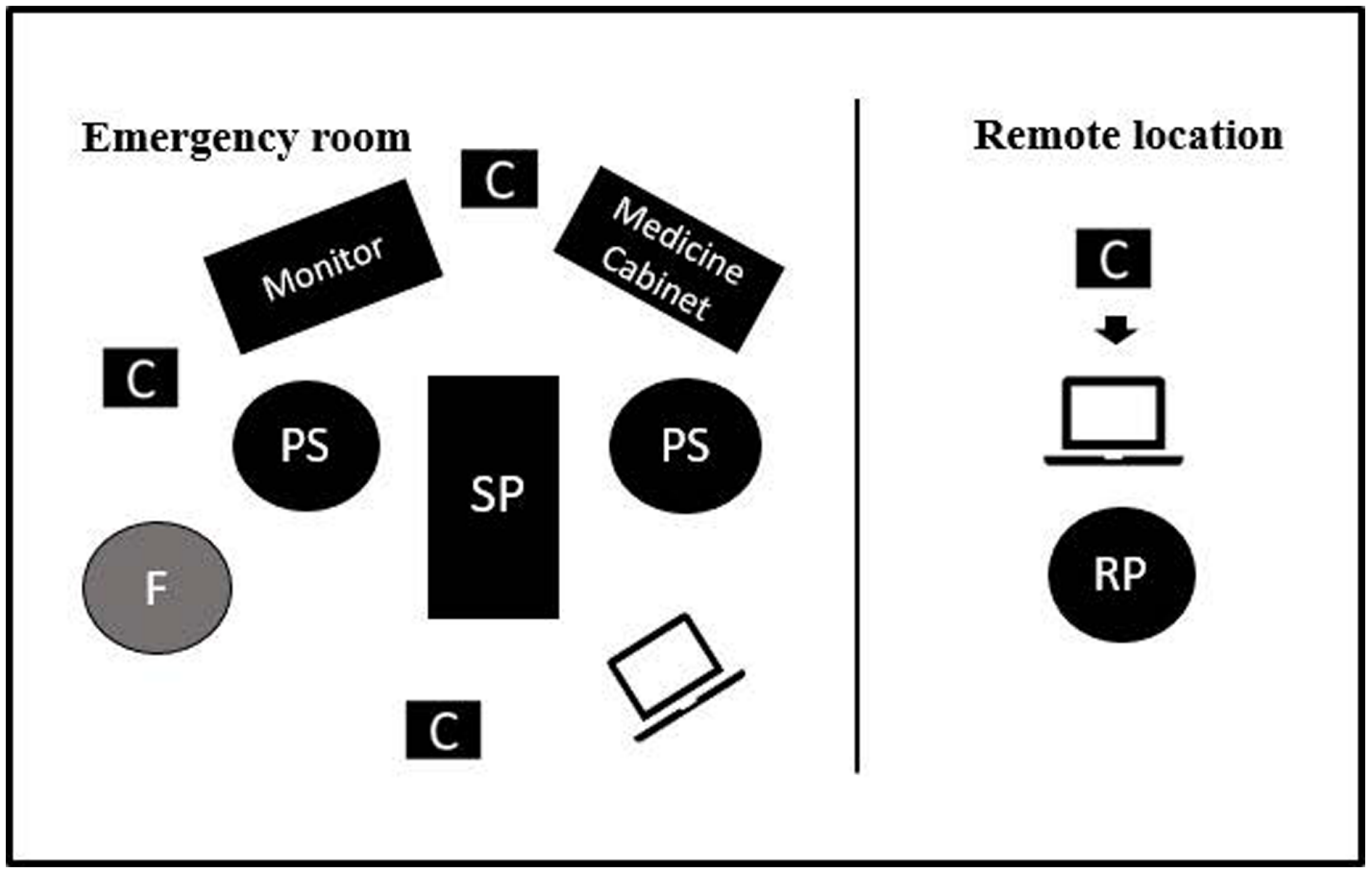
Setup for simulation team training. SP, Standardized patient; PS, Proximal staff (assistant nurse, registered nurse, or student nurses); RP, Remote physician or remote medical student; F, Facilitator; C, Camera. In the remote location, the camera was integrated into the laptop. The laptop in the emergency room was on a portable table, so the figure demonstrates a typical but not fixed location. Monitor for patient vital signs. Medicine cabinet contained emergency medical equipment.

**Figure 2:**
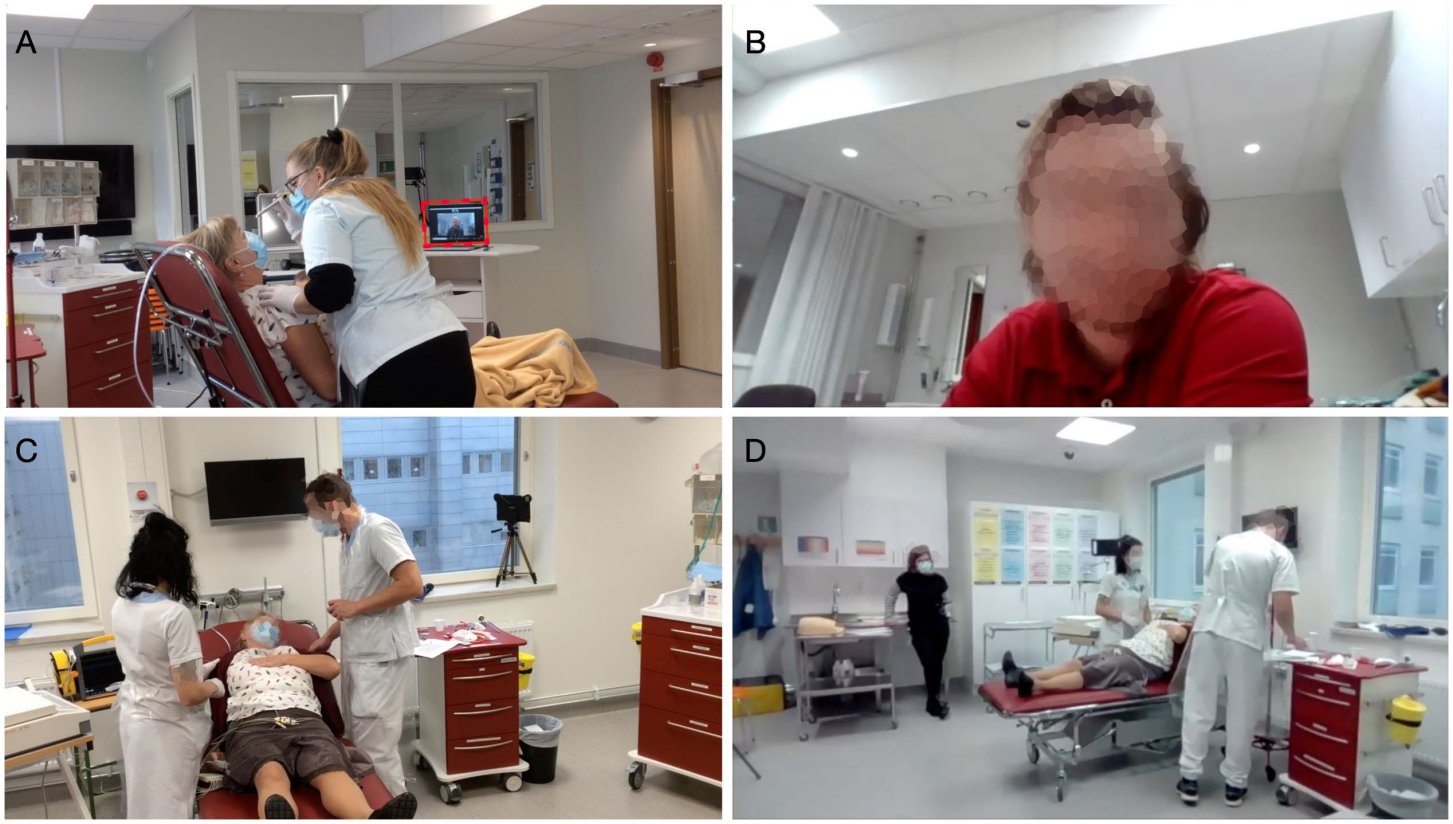
Different camera views. **(A)** Standardized patient and proximal staff; laptop screen displays the remote physician, encircled in red. **(B)** Remote physician. **(C)** Proximal staff and standardized patient. **(D)** Remote physician’s camera view: facilitator, proximal staff, and standardized patient.

To emphasize the location of the participants during the simulation-based team training, the student nurses, assistant nurses, and RNs are referred to herein as *proximal staff*, since they were located in the emergency room with the patient. The medical students and physicians are referred to as *remote physicians*, since they participated in a separate room. A setup with the common locations of the participants during the simulation-based team training is illustrated in Figure 1. In both locations (i.e., the emergency room and the remote room), there were laptops equipped with Zoom™ video conferencing software—that is, a synchronous audiovisual communication platform—for connectivity. When the proximal staff needed to consult a remote physician, they initiated contact through the platform. An external loudspeaker amplified the sound of the connection in the emergency room. The laptop in the emergency room was placed on a portable table, facilitating direct interaction with the patient. A vital sign monitor—which is typically used during patient care—displaying the patient’s heart rate, blood pressure, and peripheral oxygen saturation was present and facilitator-controlled for further simulation authenticity. The facilitator was present in the room’s periphery while the scenario was running to provide information on clinical tests that the standardized patient could not display. To allow for the later assessment of team performance, the proximal staff were audio- and video-recorded from different angles, and the remote physician was recorded through the live-video feed. The setup, including camera views, is presented in Figure 2.

##### 2.2.1.4. Implementation of simulation-based team training

Before the simulated team training scenarios started, the participants viewed a 10-min video describing patient safety and teamwork according to the Crew Resource Management (CRM) concept (Helmreich et al., 1999) and initial assessment and treatment based on the Advanced Trauma Life Support (ATLS) program (American College of Surgeons Committee on Trauma, 2013). The research group made the introduction video.

Then, the participants were informed about the training structure, available resources and equipment, and function of the educational staff. The participants reported their basic demographics through a questionnaire regarding their age, gender, medical education, and work experience. In addition, questions were asked about previous experience in team training and prior experience working in distributed medical teams relying on synchronized communication technology (Table 1).

**Figure 3:**
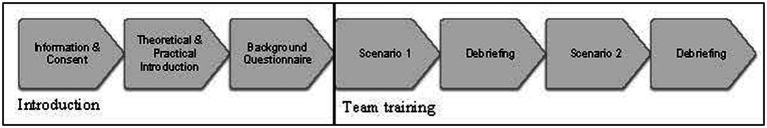
Flowchart for Stage 1: simulation-based team training. The time required for information and consent, theoretical and practical introduction, and background questionnaire was 1 h. Each scenario was designed to last for about 20 min, and debriefing was carried out for 15 min.

The team training sessions started with a facilitator-led briefing on the primary goal of the training sessions. The participants were encouraged to use all available resources in the team and to use a systematic approach to treat the patient. The scenarios then started with a handover from the facilitator, who gave brief background information on the patient. Team members were instructed to follow standardized operating procedures and medical guidelines to identify the medical condition and start necessary treatments. The facilitator ended the scenario when the team stabilized the patient’s vital signs and communicated a diagnosis and continued care plan. The facilitator initiated and led a debriefing that focused on medical treatment (task performance) and teamwork skills, which lasted for about 15 min, immediately after completing each scenario. Figure 3 provides a flowchart of the simulation-based team training in Stage 1.

#### 2.2.2. Stage 2: rating procedure

Data was collected in spring of 2022. Three raters assessed video-recorded simulation-based team training.

##### 2.2.2.1. Participants

We recruited three raters with a convenience sample for rating procedures with the TEAM instrument. Rater 1 was a critical care registered nurse and PhD in nursing. Rater 2 was a consultant physician in anesthesia and intensive care and PhD student. Rater 3 was a resident physician in anesthesia and intensive care medicine. Raters 1 and 2 had more than 12 years of experience as simulation facilitators and raters, while Rater 3 was a novice. Both genders were represented.

##### 2.2.2.2. Rating procedure

**Figure 4:**
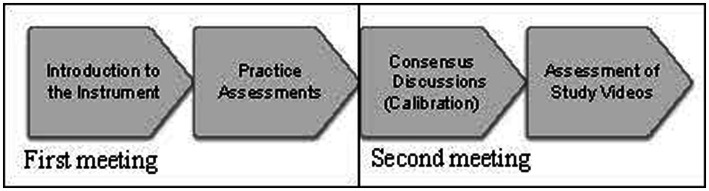
Flowchart for Stage 2: rating procedure. At the first meeting, the instrument was introduced for 2 h. Then, during 2 weeks, the raters assessed two scenarios of 20 min each. In the second meeting, a consensus discussion took place for 2 h. Within 2 months, all raters individually assessed 18 video-recorded simulation-based team training sessions.

The raters were introduced to the TEAM instrument in its original version and an additional guide (Cooper et al., 2010). As a preparation, the raters independently practiced assessments on two video-recorded team training simulations (2 × 20 min) equivalent to those included in the study. Then, the raters discussed their scores to establish a common understanding of the instrument and reach a consensus (calibration) for the rating procedure. The video-recorded scenarios (*n* = 18) were coded, and the assessments were assigned randomly. Figure 4 provides a flowchart of the rating process in Stage 2.

##### 2.2.2.3. The instrument

TEAM is an item-based instrument for assessing teamwork developed by Cooper et al. and composed of three domains: *leadership* (items 1–2), *teamwork* (items 3–9), and *task management* (items 10–11; Cooper et al., 2010; Cooper, 2022). Each item is rated on a five-point Likert scale, ranging from 0 = Never/hardly ever to 4 = Always/nearly always, based on the frequency of occurrence of defined behaviors. In total, the maximum score is 44. According to Cooper et al., scores of 33 or less, 34–39, and 40–44 indicate poor, good, and excellent team performance, respectively. In addition, a twelfth item, *overall performance,* is rated on a scale of 1–10, based on the rater’s overall “gut reaction” to the global team performance. Global rating scores below 7 indicate poor performance, while 9–10 are considered excellent.

### 2.3. Data analysis

Based on previous work (Hultin et al., 2019), a sample size of nine teams was suggested. For inter-rater reliability calculations, Koo et al. recommend three raters (Koo and Li, 2016). In this study, all three raters assessed all video-recorded scenarios (*n* = 54). No rating data was missing. SPSS Statistics for Windows version 28 (IBM, 2021) was used to compute descriptive statistics and the validity and reliability outcomes.

This study was methodologically guided in reliability and validity, based on the definitions of these terms by Streiner et al. (2015). *Reliability* assesses that the instrument measures something in a reproducible fashion; in other words, it is the extent to which a research instrument consistently has the same results if used in the same situation repeatedly. However, reliability says nothing about what is being measured; valid evidence is required to determine that. *Validation* is a process of determining what concept is being accurately measured with the instrument (Streiner et al., 2015).

*Inter-rater reliability* using intraclass correlation (ICC) explores the variation between raters when assessing the same group of subjects. With guidance from Koo et al., we calculated the ICC based on the model: two-way random effect; type: average measure; and definition: consistency and absolute agreement (Koo and Li, 2016). Calculations were made on TEAM domains using the mean score of each rater and on each item using each rater’s scoring results. The two-way random effect regards the raters as randomly selected from a larger population with similar characteristics. Average measures were chosen, since the data were based on the mean of multiple raters. Analyses of both consistency and absolute agreement were made, because we intended to measure whether the raters’ scores for the same group of subjects were correlated in an additive manner (consistency) and whether different raters assigned the same score to the same subject (absolute agreement) *Internal consistency*, which explores the extent to which all items measure the same concept (Tavakol and Dennick, 2011), was calculated using Cronbach’s alpha on the mean value of the raters for the items in each of the three TEAM domains. Cronbach’s alpha was also measured on all TEAM items except item 12. *Predictive validity* is the extent to which the instrument’s results predict the outcome (Streiner et al., 2015). To reflect the variation in TEAM scores across the teams’ experience levels and between scenarios, a one-way analysis of variance, the Kruskal Wallis test, was calculated with an exact *p* value. The mean value for the respective rater scores in each TEAM domain was compared with the proficiency levels (beginner, intermediate, and expert) and scenarios (urosepsis and myocardial infarction). Finally, *concurrent validity* shows the extent of the agreement between two measures or assessments taken at the same time (Streiner et al., 2015). Using Pearson’s method, correlations were calculated between the overall performance scores and the three TEAM domains. A statistical significance was considered with a *p* value <0.05.

## 3. Results

### 3.1. Reliability

**Figure 5:**
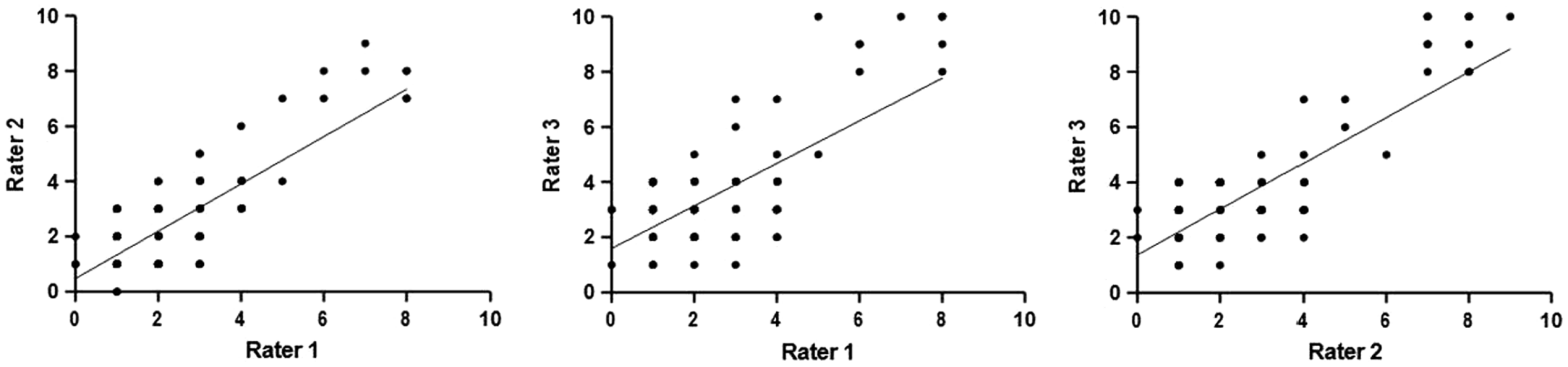
Item rating correlation between the raters, as fitted by linear regression. Rater 1 vs. Rater 2: *Y* = 0.8572*X + 0.4800, *p* < 0.0001. Rater 1 vs. Rater 3: *Y* = 0.7714*X + 1.595, *p* < 0.0001. Rater 2 vs. Rater 3: *Y* = 0.8284*X + 1.376, *p* < 0.0001. The *p*-value is the significance for the slope being non-zero; i.e., a correlation between *x* and *y*.

The ICC values for the domains and item levels are presented in Table 2. For consistency, the ICC (95% confidence interval) calculated for the TEAM domains of leadership, teamwork, and task management were 0.74 (0.42–0.89), 0.92 (0.81–0.97), and 0.85 (0.67–0.94), respectively. According to Koo et al., these values correspond to moderate, excellent, and good inter-rater reliability in the respective domains (Koo and Li, 2016). For absolute agreement, the corresponding values for leadership, teamwork, and task management were 0.59 (0.10–0.83), 0.82 (0.36–0.94), and 0.78 (0.46–0.92), respectively, indicating good reliability. Moreover, the ICCs for item 12 were 0.91 (0.81–0.97) and 0.80 (0.30–0.94), regarding consistency and absolute agreement, indicating excellent and good inter-rater reliability, respectively, as the overall rating. The item rating correlation between raters was fitted by linear regression, as shown in Figure 5.

**Table 2:** Intraclass correlation.

Items in TEAM	ICC (CI 95%)	ICC (CI 95%)
	Consistency	Absolute agreement
Leadership	0.74 (0.42–0.89)	0.59 (0.10–0.83)
1. The team leader let the team know what was expected of them through direction and command.	0.65 (0.24**–**0.86)	0.55 (0.09**–**0.81)
2. The team leader maintained a global perspective.	0.70 (0.34**–**0.88)	0.54 (0.05**–**0.81)
Teamwork	0.92 (0.81–0.97)	0.82 (0.36–0.94)
3. The team communicated effectively.	0.64 (0.20**–**0.85)	0.64 (0.21**–**0.85)
4. The team worked together to complete the tasks in a timely manner.	0.93 (0.84**–**0.97)	0.89 (0.71**–**0.96)
5. The team acted with composure and control.	0.47 (0**–**0.78)	0.36 (0**–**0.71)
6. The team morale was positive.	0.80 (0.56**–**0.92)	0.72 (0.35**–**0.89)
7. The team adapted to changing situations.	0.87 (0.71**–**0.95)	0.73 (0.22**–**0.91)
8. The team monitored and reassessed the situation.	0.83 (0.62**–**0.93)	0.72 (0.30**–**0.89)
9. The team anticipated potential actions.	0.78 (0.51**–**0.91)	0.73 (0.42**–**0.89)
Task management	0.85 (0.67–0.94)	0.78 (0.46–0.92)
10. The team prioritized tasks.	0.79 (0.54**–**0.92)	0.78 (0.53**–**0.91)
11. The team followed approved standards/guidelines.	0.76 (0.46**–**0.90)	0.62 (0.14**–**0.85)
**Overall**		
12. On a scale of 1–10, give your global rating of the team’s performance.	0.91 (0.81**–**0.97)	0.80 (0.30**–**0.94)

### 3.2. Internal consistency

The internal consistency measured with Cronbach’s alpha for the three TEAM domains (leadership, teamwork, and task management) were 0.94, 0.97, and 0.89, respectively, indicating excellent internal consistency. For the total scores of items 1–11, Cronbach’s alpha was 0.97.

### 3.3. Predictive validity

**Figure 6:**
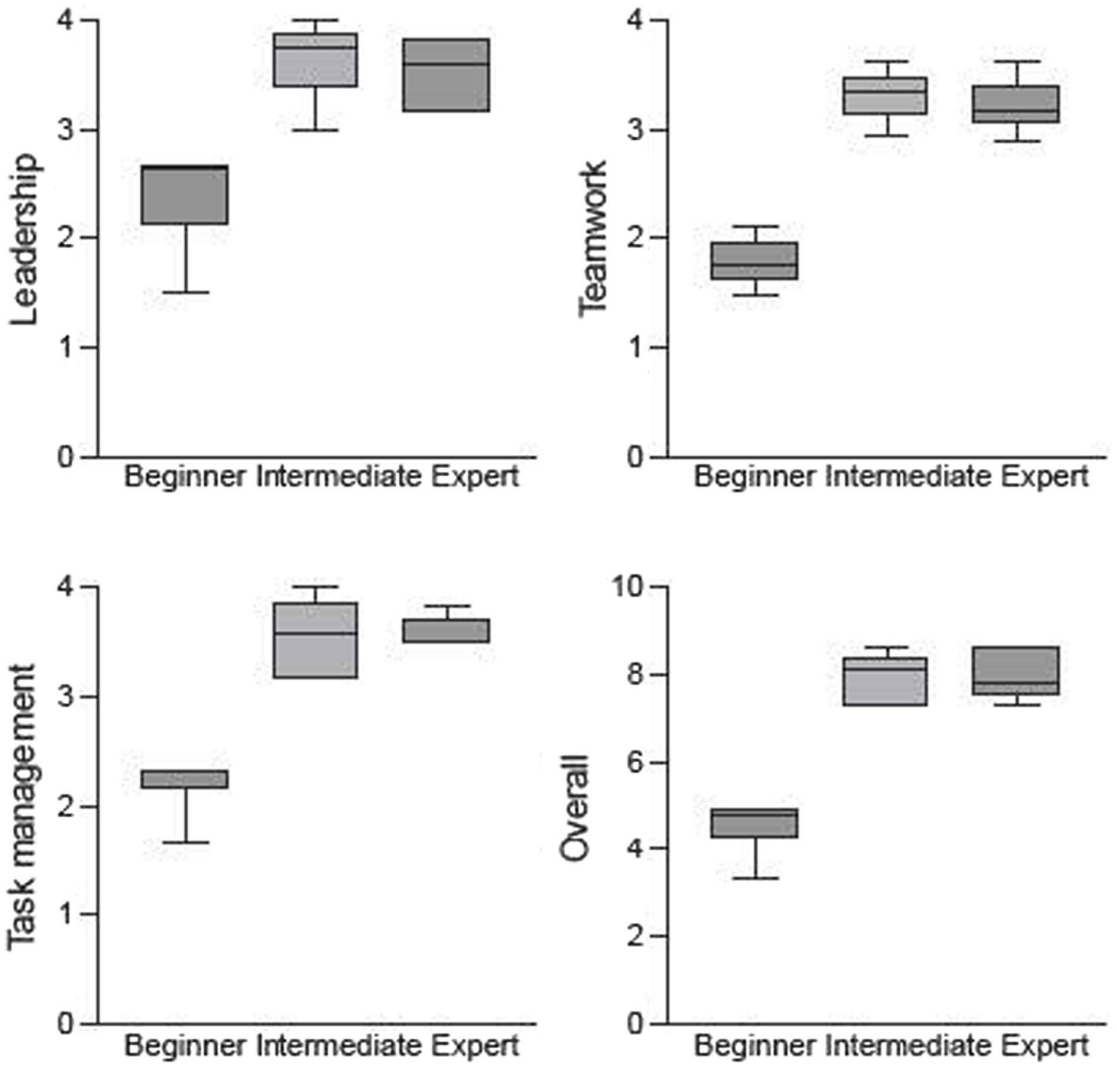
Boxplots showing the distribution of the ratings for TEAM domains and overall performance depending on proficiency levels. In all TEAM domains, the scores significantly correlated with the proficiency level (*p*-value < 0.001 for all dimensions).

In all three TEAM domains, as well as in the overall performance, significant differences were found between the beginner, intermediate, and expert groups in terms of the performances scores (*p* < 0.001). The boxplots in Figure 6 illustrate the main differences between the beginners and the other two team categories (intermediates and experts). A comparison of the three TEAM domains for the two scenarios (urosepsis vs. myocardial infarction) showed no significant difference.

### 3.4. Concurrent validity

Finally, there was a positive correlation between the scores in the three TEAM domains and the 12th item (overall), all of which were above 0.92. This indicates a strong connection between the concept’s leadership, teamwork, task management, and overall team performance.

## 4. Discussion

This study aimed to test the validity and reliability of TEAM in distributed healthcare teams working in an acute simulated setting. Overall, we found strong inter-rater reliability and internal consistency, suggesting that TEAM can be used to assess team performance with a remote physician. New demands from a continuously changing workplace emphasize the importance of development in team research. Today, there is a need to investigate alternative team structures and to understand better what it means to connect short-lasting *ad hoc* emergency teams and technology (White et al., 2018). Since non-technical skills contribute to providing safe patient care and positively influencing the quality of teamwork (Cooper et al., 2016), developing team training adapted to distributed team settings and their different working conditions is essential.

Good psychometric properties have previously been reported for the TEAM instrument in terms of validity and reliability in various settings for co-located teams (Cooper et al., 2010, 2016; Maignan et al., 2016; Hultin et al., 2019). However, physically separating the team profoundly affects the prerequisites for teamwork (Butler et al., 2019). Therefore, the reliability and validity of the instrument could not be taken for granted in this alternate setting.

According to Shoukri et al., the estimated ICC value depends on the sampled subjects’ heterogeneity; in other words, the more heterogeneity, the higher the ICC value (Shoukri et al., 2004). Low values may reflect a lack of variability in subjects, a small number of issues, or a small number of raters. Freytag et al. compared novice and expert raters using TEAM in simulated emergencies and found a similar distribution of the ratings, even though the novices were slightly more forgiving in rating behavior (Freytag et al., 2019). In this study, the ICC values of the three raters with somewhat different backgrounds and experiences were consistent.

The values for Cronbach’s alpha were between 0.89–0.97. An alpha value greater than 0.7 is considered acceptable, even though values above 0.8 are preferable. Nevertheless, values >0.90 might not be desirable unless in high-stakes examinations, as may indicate item redundancy (Tavakol and Dennick, 2011). The original version of TEAM was validated for a cardiac resuscitation context (Cooper et al., 2010). The values for Cronbach’s alpha in that context were 0.89, and a validation of a Swedish translation of TEAM yielded alpha values of 0.96 (Karlgren et al., 2021). Our findings on internal consistency are similarly high, indicating that some items might be redundant. As instrument development was not the topic of this study, we only conclude that the instrument has excellent internal consistency in this setting.

Having teams with different proficiency levels allowed us to test the scale for predictive validity. We found that the beginner groups’ scores differed from those of the intermediate and expert groups. There were minor differences between the intermediates and experts, reflecting the difficulty in assessing the experience and knowledge of the already-established staff. Furthermore, younger and less clinically experienced staff may have more experience in electronically mediated communication. In this study, some of the participants at the beginner level had previous experience in distributed settings. The medical staff at the expert levels were experts in clinical expertise but not necessarily in teamwork *via* video communication. Moreover, it is well known that familiarity among team members positively affects communication and performance (Marlow et al., 2018). In our study, in contrast to participants at the beginner level, intermediates and experts were familiar with each other, which could contribute to the results on predictive validity. Hence these results should be interpreted with caution. No significant differences were found between the TEAM domains and the scenarios. This could result from the work done beforehand to make the scenarios equally complex.

According to von Wendt et al., the most crucial factor in a scientific investigation is deciding on an instrument suited for the research and field of study (von Wendt and Niemi-Murola, 2018). Also, Schmutz et al. claim that there is no “one-size-fits-all training method” and that factors such as norms and collaboration influence teamwork and clinical performance (Schmutz et al., 2019). Although they had varying backgrounds and levels of experience, the raters in this study perceived the TEAM instrument as user-friendly with a clear design. The raters in this study used the original version of the instrument, since they had used it before; moreover, by validating the English version, access to a larger community of users is provided. Karlgren et al. translated and validated the TEAM instrument in Swedish and struggled with the first leadership item, since “through command” was considered to be culturally inappropriate in Swedish healthcare (Karlgren et al., 2021). The wording was negatively perceived as being authoritarian. In our preparatory work, when the raters were gaining a shared sense of the instrument, we reasoned along the same lines as Karlgren et al. “that team leaders should convey a plan to the team” rather than give command (Karlgren et al., 2021).

## 5. Limitations

We acknowledge that this study has some limitations. When conducting a reliability study, Koo et al. suggest at least 30 heterogeneous samples and a minimum of three raters (Koo and Li, 2016). The ICC values we obtained for the TEAM instrument aligned with those reported by previous researchers (Cooper et al., 2010; Carpini et al., 2021; Karlgren et al., 2021). However, due to the small sample size (*n* = 9) in this study, some caution is warranted regarding our findings on reliability. For this type of study, three raters may be regarded as acceptable, and using video recordings allows for double-checked observations and access to the same camera views (Karlgren et al., 2021). Due to the recruitment strategy, the sample of participants for the team training was not controlled for. It is possible that some of the groups contained particularly motivated and high-performing individuals; however, this situation is likely to have been similar for all the groups, independent of the proficiency level. Another limitation of this study is its simulated environment, which may not fully represent the complexity of the real-world setting. However, the scenarios were scripted with commonly occurring emergencies within the setting of a rural primary care healthcare center that relies on a distributed team. Future research could add to our findings with ratings from actual emergencies in distributed settings, thereby strengthening transferability.

## 6. Conclusion

In conclusion, when tested in a distributed team setting, TEAM was found to be a valid and reliable instrument for assessing emergency medical teamwork. This finding indicates that the instrument is feasible for use when assessing non-technical skills for providing safe care in distributed teams. To the best of our knowledge, the instrument had not been previously validated in this context. Our findings can help focus future-directed learning in healthcare and assist future research on distributed healthcare teams.

## Data availability statement

The original contributions presented in the study are included in the article/supplementary material, further inquiries can be directed to the corresponding author.

## Ethics statement

The studies involving human participants were reviewed and approved by the Swedish Ethical Review Authority. The participants provided their written informed consent to participate in this study. Written informed consent was obtained from the individual(s) for the publication of any potentially identifiable images or data included in this article.

## Author contributions

JC obtained funding for the study. The study was conceived by JC, MHä, and MHu. HM, MHä, KJ, and TN participated in data collection. HJ, HM, JC, MHä, and MHu planned the statistical analysis and interpreted the data. HM carried out the formal analysis and the first draft preparation. HM, MHä, MHu, HJ, KJ, TN, and JC edited the manuscript. All authors contributed to the article and approved the submitted version.

## Funding

This study was supported by unrestricted funding from the Kamprad Family Foundation for Entrepreneurship, Research and Charity and LÖF—The National Swedish Patient Company.

## Conflict of interest

The authors declare that the research was conducted in the absence of any commercial or financial relationships that could be construed as a potential conflict of interest.

## Publisher’s note

All claims expressed in this article are solely those of the authors and do not necessarily represent those of their affiliated organizations, or those of the publisher, the editors and the reviewers. Any product that may be evaluated in this article, or claim that may be made by its manufacturer, is not guaranteed or endorsed by the publisher.

## References

[ref1] American College of Surgeons Committee on Trauma (2013). Advanced trauma life support (ATLS®): the ninth edition. J. Trauma Acute Care Surg. 74, 1363–1366. doi: 10.1097/01586154-201305000-00026, PMID: 23609291

[ref2] Bains J. Greenwald P. W. Mulcare M. R. Leyden D. Kim J. Shemesh A. J.. (2020). Utilizing telemedicine in a novel approach to COVID-19 management and patient experience in the emergency department. Telemed. J. E Health 27, 254–260. doi: 10.1089/tmj.2020.0162 32821027

[ref3] Bell B. Kozlowski S. A. (2002). Typology of virtual teams: implications for effective leadership. Group Org. Manag. 27, 14–49. doi: 10.1177/1059601102027001003

[ref4] Bhangu A. Stevenson C. Szulewski A. MacDonald A. Nolan B. (2022). A scoping review of nontechnical skill assessment tools to evaluate trauma team performance. J. Trauma Acute Care Surg. 92, e81–e91. doi: 10.1097/TA.0000000000003492, PMID: 34908024

[ref5] Boet S. Etherington N. Larrigan S. Yin L. Khan H. Sullivan K.. (2019). Measuring the teamwork performance of teams in crisis situations: a systematic review of assessment tools and their measurement properties. BMJ Qual. Saf. 28, 327–337. doi: 10.1136/bmjqs-2018-008260, PMID: 30309910

[ref6] Bolle S. R. Larsen F. Hagen O. Gilbert M. (2009). Video conferencing versus telephone calls for team work across hospitals: a qualitative study on simulated emergencies. BMC Emerg. Med. 9. doi: 10.1186/1471-227X-9-22, PMID: 19943978 PMC2794251

[ref7] Butler L. Whitfill T. Wong A. H. Gawel M. Crispino L. Auerbach M. (2019). The impact of telemedicine on teamwork and workload in pediatric resuscitation: a simulation-based, randomized controlled study. Telemed. J. E Health 25, 205–212. doi: 10.1089/tmj.2018.0017, PMID: 29957150

[ref8] Cant R. P. Porter J. E. Cooper S. J. Roberts K. Wilson I. Gartside C. (2016). Improving the non-technical skills of hospital medical emergency teams: the TEAM emergency assessment measure (TEAM). Emerg. Med. Australas. 28, 641–646. doi: 10.1111/1742-6723.12643, PMID: 27474369

[ref9] Carpini J. A. Calvert K. Carter S. Epee-Bekima M. Leung Y. (2021). Validating the TEAM emergency assessment measure (TEAM) in obstetric and gynaecologic resuscitation teams. Aust. N. Z. J. Obstet. Gynaecol. 61, 855–861. doi: 10.1111/ajo.13362, PMID: 33908031

[ref10] Cooper S. (2022). TEAM. Available at: https://medicalemergencyteam.com/members-area/ (Accessed 28 November 2022).

[ref11] Cooper S. Cant R. Connell C. Sims L. Porter J. E. Symmons M.. (2016). Measuring teamwork performance: validity testing of the TEAM emergency assessment measure (TEAM) with clinical resuscitation teams. Resuscitation 101, 97–101. doi: 10.1016/j.resuscitation.2016.01.026, PMID: 26875992

[ref12] Cooper S. Cant R. Porter J. Sellick K. Somers G. Kinsman L.. (2010). Rating medical emergency teamwork performance: development of the TEAM emergency assessment measure (TEAM). Resuscitation 81, 446–452. doi: 10.1016/j.resuscitation.2009.11.027, PMID: 20117874

[ref13] Craig J. Patterson V. (2005). Introduction to the practice of telemedicine. J. Telemed. Telecare 11, 3–9. doi: 10.1177/1357633X0501100102, PMID: 15829036

[ref14] Felix H. M. Simon L. V. (2020). Types of standardized patients and recruitment in medical simulation. *StatPearls*. Treasure Island (FL): StatPearls Publishing. 31751097

[ref15] Field M. J. (1996). Telemedicine: A guide to assessing telecommunications in health care, Washington (DC): The National Academies Press. 20845554

[ref16] Freytag J. Stroben F. Hautz W. E. Schauber S. K. Kammer J. E. (2019). Rating the quality of teamwork-a comparison of novice and expert ratings using the TEAM emergency assessment measure (TEAM) in simulated emergencies. Scand. J. Trauma Resusc. Emerg. Med. 27:12. doi: 10.1186/s13049-019-0591-9, PMID: 30736821 PMC6368771

[ref17] Garattini L. Badinella Martini M. Mannuccio Mannucci P. (2021). Improving primary care in Europe beyond COVID-19: from telemedicine to organizational reforms. Intern. Emerg. Med. 16, 255–258. doi: 10.1007/s11739-020-02559-x, PMID: 33196973 PMC7668282

[ref18] Helmreich R. L. Merritt A. C. Wilhelm J. A. (1999). The evolution of crew resource management training in commercial aviation. Int. J. Aviat. Psychol. 9, 19–32. doi: 10.1207/s15327108ijap0901_2, PMID: 11541445

[ref19] Hultin M. Jonsson K. Hargestam M. Lindkvist M. Brulin C. (2019). Reliability of instruments that measure situation awareness, team performance and task performance in a simulation setting with medical students. BMJ Open 9:e029412. doi: 10.1136/bmjopen-2019-029412, PMID: 31515425 PMC6747650

[ref20] IBM. (2021). IBM SPSS statistics for windows: Version 28.0. Armonk, NY: IBM Corp.

[ref21] Karlgren K. Dahlstrom A. Birkestam A. Drevstam Norling A. Forss G. Andersson Franko M.. (2021). The TEAM instrument for measuring emergency team performance: validation of the Swedish version at two emergency departments. Scand. J. Trauma Resusc. Emerg. Med. 29:139. doi: 10.1186/s13049-021-00952-9, PMID: 34544459 PMC8454124

[ref22] Kohn L. Corrigan J. (2000). To err is human: Building a safer health system. Washington, D.C: National Academy Press. 25077248

[ref23] Koo T. K. Li M. Y. (2016). A guideline of selecting and reporting Intraclass correlation coefficients for reliability research. J. Chiropr. Med. 15, 155–163. doi: 10.1016/j.jcm.2016.02.012, PMID: 27330520 PMC4913118

[ref24] Maignan M. Koch F. X. Chaix J. Phellouzat P. Binauld G. Collomb Muret R.. (2016). TEAM emergency assessment measure (TEAM) for the assessment of non-technical skills during resuscitation: validation of the French version. Resuscitation 101, 115–120. doi: 10.1016/j.resuscitation.2015.11.024, PMID: 26708450

[ref25] Manser T. (2009). Teamwork and patient safety in dynamic domains of healthcare: a review of the literature. Acta Anaesthesiol. Scand. 53, 143–151. doi: 10.1111/j.1399-6576.2008.01717.x, PMID: 19032571

[ref26] Marlow S. L. Lacerenza C. N. Paoletti J. Burke S. Salas E. (2018). Does team communication represent a one-size-fits-all approach?: a metaanalysis of team communication and performance. Organ. Behav. Hum. Decis. Process. 144, 145–170. doi: 10.1016/j.obhdp.2017.08.001

[ref27] Mickan S. M. Rodger S. A. (2005). Effective health care teams: a model of six characteristics developed from shared perceptions. J. Interprof. Care 19, 358–370. doi: 10.1080/13561820500165142, PMID: 16076597

[ref28] O'Leary M. B. Cummings J. N. (2007). The spatial, temporal, and configurational characteristics of geographic dispersion in teams. MIS Q. 31, 433–452. doi: 10.2307/25148802

[ref29] Salas E. Sims D. E. Burke C. S. (2016). Is there a “big five” in teamwork? Small Group Res. 36, 555–599. doi: 10.1177/1046496405277134

[ref30] Schmutz J. B. Meier L. L. Manser T. (2019). How effective is teamwork really? The relationship between teamwork and performance in healthcare teams: a systematic review and meta-analysis. BMJ Open 9:e028280. doi: 10.1136/bmjopen-2018-028280, PMID: 31515415 PMC6747874

[ref31] Shapiro M. J. Gardner R. Godwin S. A. Jay G. D. Lindquist D. G. Salisbury M. L.. (2008). Defining team performance for simulation-based training: methodology, metrics, and opportunities for emergency medicine. Acad. Emerg. Med. 15, 1088–1097. doi: 10.1111/j.1553-2712.2008.00251.x, PMID: 18828830

[ref32] Shoukri M. M. Asyali M. H. Donner A. (2004). Sample size requirements for the design of reliability study: review and new results. Stat. Methods Med. Res. 13, 251–271. doi: 10.1191/0962280204sm365ra

[ref33] Streiner D. L. Norman G. R. Cairney J. (2015). Health measurment scales: A practical guide to their development and use. 5th *Edn*. New York: Oxford University Press.

[ref34] Tavakol M. Dennick R. (2011). Making sense of Cronbach's alpha. Int. J. Med. Educ. 2, 53–55. doi: 10.5116/ijme.4dfb.8dfd, PMID: 28029643 PMC4205511

[ref35] Valentine M. A. Nembhard I. M. Edmondson A. C. (2015). Measuring teamwork in health care settings a review of survey instruments. Med. Care 53, e16–e30. doi: 10.1097/MLR.0b013e31827feef6 24189550

[ref36] Vilendrer S. Patel B. Chadwick W. Hwa M. Asch S. Pageler N.. (2020). Rapid deployment of inpatient telemedicine in response to COVID-19 across three health systems. J. Am. Med. Inform. Assoc. 27, 1102–1109. doi: 10.1093/jamia/ocaa077, PMID: 32495830 PMC7314045

[ref37] von Wendt C. E. A. Niemi-Murola L. (2018). Simulation in Interprofessional clinical education: exploring validated nontechnical skills measurement tools. Simul. Healthc. 13, 131–138. doi: 10.1097/SIH.0000000000000261 29117089

[ref38] Weaver S. J. Dy S. M. Rosen M. A. (2014). Team-training in healthcare: a narrative synthesis of the literature. BMJ Qual. Saf. 23, 359–372. doi: 10.1136/bmjqs-2013-001848, PMID: 24501181 PMC3995248

[ref39] White B. A. A. Eklund A. McNeal T. Hochhalter A. Arroliga A. C. (2018). Facilitators and barriers to ad hoc team performance. Proc (Bayl Univ Med Cent) 31, 380–384. doi: 10.1080/08998280.2018.1457879, PMID: 29904320 PMC5997057

[ref40] Yule S. Flin R. Maran N. Rowley D. Youngson G. Paterson-Brown S. (2008). Surgeons' non-technical skills in the operating room: reliability testing of the NOTSS behavior rating system. World J. Surg. 32, 548–556. doi: 10.1007/s00268-007-9320-z, PMID: 18259809

